# Exploration of the Mechanisms of Differential Indole Alkaloid Biosynthesis in Dedifferentiated and Cambial Meristematic Cells of *Catharanthus roseus* Using Transcriptome Sequencing

**DOI:** 10.3389/fgene.2022.867064

**Published:** 2022-06-30

**Authors:** Pengfei Zhou, Mingxiang Chen

**Affiliations:** ^1^ School of Basic Medical Science, Guangdong Medical University, Dongguan, China; ^2^ School of Pharmacy, Guangdong Medical University, Dongguan, China

**Keywords:** indole alkaloid biosynthesis, MAPK signaling, periwinkle, plant-hormone signaling, tissue culture, vindoline, vinblastine

## Abstract

*Catharanthus roseus* produces terpenoid indole alkaloids (TIAs) of high medicinal importance. The current research focuses on finding an efficient production system such as cell suspension cultures for high TIA concentrations. *Catharanthus roseus* cambial meristematic cells (CMCs) offer multiple advantages over dedifferentiated cells (DDCs) regarding growth, homogeneity, and shear resistance. Our lab has established a CMC culture system induced by *C. roseus* cambium. We determined the concentrations of TIAs in CMCs and DDCs. CMCs produced significantly higher concentrations of total alkaloids, vindoline, vinblastine, catharanthine, and ajmalicine as compared to DDCs. We then performed Illumina HiSeq transcriptome sequencing of CMCs and DDCs and explored the differential transcriptomic signatures. Of the 96,004 unigenes, 9,564 were differentially expressed between the 2 cell suspension types. These differentially expressed genes (DEGs) were enriched in 137 KEGG pathways. Most importantly, genes from the indole alkaloid biosynthesis and the upstream pathways i.e., tryptophan metabolism, monoterpenoid biosynthesis, tropane, piperidine, and pyridine alkaloid biosynthesis, and terpenoid backbone biosynthesis showed differential transcriptomic signatures. Remarkably, the expression of genes associated with plant hormone biosynthesis, signaling, and MAPK signaling pathways was relatable to the different TIA concentrations in CMCs and DDCs. These results put forward multiple target genes, transcription factors, and regulators to develop a large-scale TIA production system using *C. roseus* CMCs.

## 1 Introduction

Kingdom Plantae is the largest untapped resource of pharmaceuticals and at least 25% of contemporary medicine is plant-based; nearly 50% of anticancer drugs are of plant origin (Fridlender et al., 2015). Many alkaloids of plant origin have been reported to show antiproliferative and anticancer effects on different types of cancers (Mondal et al., 2019). Especially vinblastine, vincristine, vindesine, and vinorelbine have been developed as anticancer drugs (Mondal et al., 2019). Periwinkle (*Catharanthus roseus*) is a rich source of terpenoid indole alkaloids (TIA) (Zhu et al., 2015). However, the TIAs are produced at low levels, which raises the production cost up to 4–60 USD per kilogram. Furthermore, the isolation process in itself is laborious (Memelink and Gantet, 2007). Continued research has highlighted that *in vitro* plant cell cultures can be an efficient replacement for the original plants. The cell cultures are more efficient, don’t need large areas (arable land) for cultivation, have lower risks associated with the environment, and could be regarded as a renewable resource.

The biosynthesis of TIAs in *C. roseus* takes place in the indole alkaloid biosynthesis pathway with at least 35 intermediates whose reactions are driven by 30 enzymes (van Der Heijden et al., 2004). All the TIAs are derived from a central precursor strictosidine (a product of the monoterpenoid biosynthesis pathway and tryptophan metabolism pathway). The biosynthesis of TIAs is involved in at least seven different subcellular compartments, thus, they can be regulated at multiple stages in different compartments. The first step (in the case of tryptophan metabolism) is the conversion of l-tryptophan to tryptamine by action of tryptophan decarboxylase (TDC). In the case of monoterpenoid biosynthesis, the reaction starts from the geranyl-pp which is converted into secologanin through a series of reactions. Geranyl-pp is converted into geraniol by the action of geranyl diphosphate diphosphatase (GES), which is then converted into secologanin by geraniol 10-hydroxylase (G10H), iridoid synthase (IRS), and loganic acid methyltransferase (LAMT). Secologanin is then converted to strictosidine under the catalysis of strictosidine synthase (STR). Next, strictosidine-*β*-d-glucosidase (SGD) is considered the key enzyme to steer the indole alkaloid biosynthesis. Further, multiple enzymes react on the SGD produced highly reactive aglycone to produce a variety of TIAs e.g., vindoline, ajmalicine, and catharanthine. The biosynthesis of TIAs in *C. roseus* has been reported to be regulated by light and plant growth regulators i.e., auxin, cytokinin, Jasmonates, and salicylic acid (SA) (Zhu et al., 2015).

*Catharanthus roseus* is one of the interesting examples of utilization of cell cultures for industrial TIA biosynthesis. However, decades of research have shown that there is a large gap to be filled regarding the production of most TIAs i.e., vinblastine and vincristine in periwinkle cell cultures is lower when compared with filed cultivation (Moreno et al., 1995; van Der Heijden et al., 2004). The development of *C. roseus* TIA production systems is an open opportunity, though in the last 4 decades, a significant amount of research has been done to develop *C. roseus* model systems for the biosynthesis and regulation of secondary metabolites. But the research failed to upscale the cell culture process and biochemical engineering the process. Thus, the current focus of the research turned to engineer the biosynthetic routes, enzymes, genes, and overall, the cell factory itself (Liu et al., 2016).

The dedifferentiated cells (DDCs) are prone to harmful genetic changes during the dedifferentiation process (Sugiyama, 2015). Furthermore, the DDCs produce heterogeneous cell types with variable TIA biosynthesis potential with slower cell growth and weaker shear resistance. On the other hand, the cambial meristematic cells (CMCs) offer unique advantages such as homogenous cell types, with faster cell growth accompanied by strong shear resistance (Moon et al., 2015). Therefore, in the long-term industrial production of TIAs through cell culture, CMCs could become a reliable production system as compared to DDCs. Additionally, different studies have reported that in DDCs certain TIAs were undetectable, thus further reducing the TIA production potential through these types of cells at a large scale (Moon et al., 2015). Another factor that researchers want to overcome is the stable production of the TIAs as their concentration tends to decrease after periodical subcultures (Neumann et al., 2012). Thus, there is a need to understand the key transcriptomic signatures that differentially regulate the TIA biosynthesis in CMCs and DDCs.

Our laboratory has established a CMC culture system induced from the cambium of periwinkle (Zhou et al., 2015; Liang et al., 2018). The periwinkle CMCs are kind of primitive and undifferentiated cells and have shown the unlimited ability of division and prolification. These cells maintain synchronous growth, keep their characteristics stable after many generations, and produce higher content of TIAs. To understand, what key transcriptomic signatures are responsible for the differential TIA biosynthesis in periwinkle CMCs and DDCs, we performed a transcriptome sequencing. Additionally, we studied the total alkaloid content, vindoline, catharanthine, ajmalicine, and vinblastine in the CMCs and DDCs by high-performance liquid chromatography (HPLC).

## 2 Results

### 2.1 Variable Content of Vindoline, Catharanthine, Ajmalicine, and Vinblastine in the CMCs and DDCs

**FIGURE 1:**
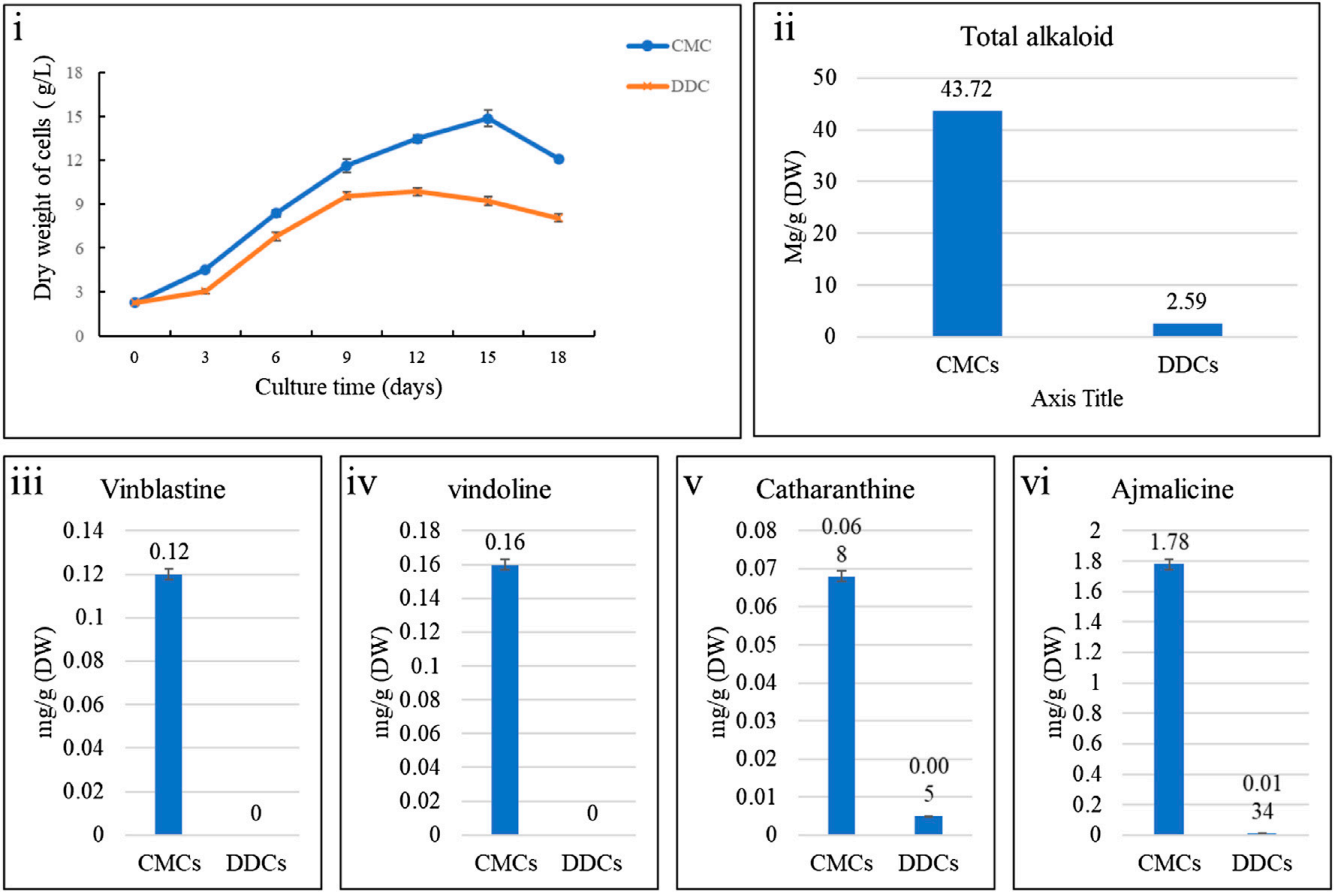
Dry weight and alkaloid content of *C. roseus* cambial meristematic cells (CMCs) and dedifferentiated cells (DDCs). i) Changes in dry weight over time, comparison of mean content of ii) total alkaloids, iii) vinblastine, iv) vindoline, v) catharanthine, and vi) ajmalicine. The bars represent mean values for three replicates. The error bars represent standard deviation.

The dry weight of the CMCs and DDCs showed variation within days. Taking same dry weight as initial material, the CMCs produced maximum dry mass on 15th day, whereas DDC produced maximum dry weight of cells on 12th day (Figure 1I). Therefore, we chose 12 days old cells to analyze the alkaloid contents. Total alkaloids, vindoline, catharanthine, ajmalicine, and vinblastine were measured in CMCs and DDCs by HPLC. Interestingly, we observed that the total alkaloid content was significantly higher in CMCs (43.72 mg/G) as compared to DDCs (2.59 mg/L) (Figure 1ii). Other indole alkaloids also showed a similar trend i.e., higher concentrations in CMCs as compared to DDCs. Vindoline and vinblastine were only detected in CMCs. CMCs had 18.77-fold and 182.57-fold higher content of catharanthine and ajmalicine as compared to DDCs, respectively (Figure 1iii–vi). These results indicate that the total alkaloid content as well as individual indole alkaloid content is higher in CMCs, thus these cells can be used for commercial alkaloid biosynthesis.

### 2.2 Transcriptome Comparison of Periwinkle CMCs and DDCs

**FIGURE 2:**
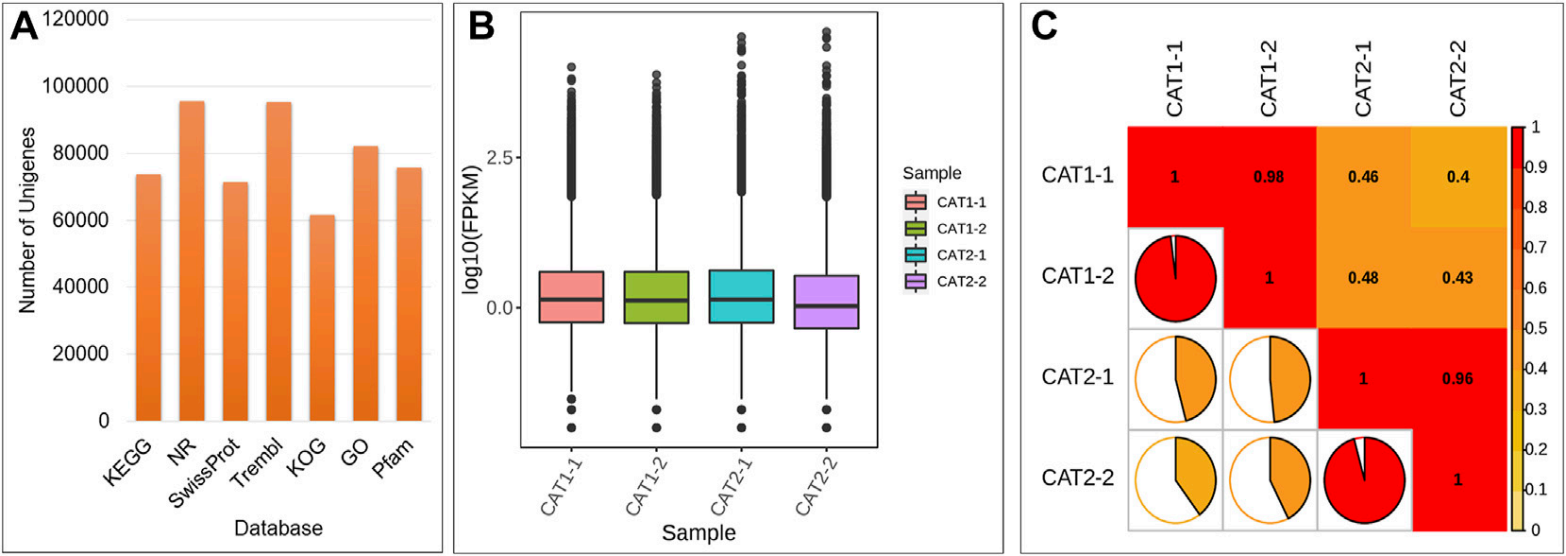
**(A)** Summary of unigene annotation in different databases, **(B)** overall distribution of sample gene expression, and **(C)** Pearson correlations between CAT-1 (CMCs) and CAT-2 (DDCs).

The Illumina HiSeq transcriptome sequencing of four samples of C. roseus cells i.e., CMCs and DDCs resulted in 50,296,033 and 46,706,898 raw and clean reads, respectively. On an average 91.33% clean reads could be mapped 42,641,912 of 46,706,898 clean reads. Overall, this project resulted in 28.02 Gb clean data; the clean data of each sample reached 6 Gb, and the Q30 base percentage was 93% and above (Supplementary Table S1). A total of 120,808 unigenes could be annotated in the seven databases; 96,004 unigenes could be annotated in at least one database (Figure 2A). The FPKM for CMCs was higher than that of DDCs (Figure 2B). Pearson Correlation Coefficient between the CMCs replicates was 0.98% whereas between the DDC replicates it was 0.96% (Figure 2C).

### 2.3 Differential Gene Expression Between Periwinkle CMCs and DDCs

**FIGURE 3:**
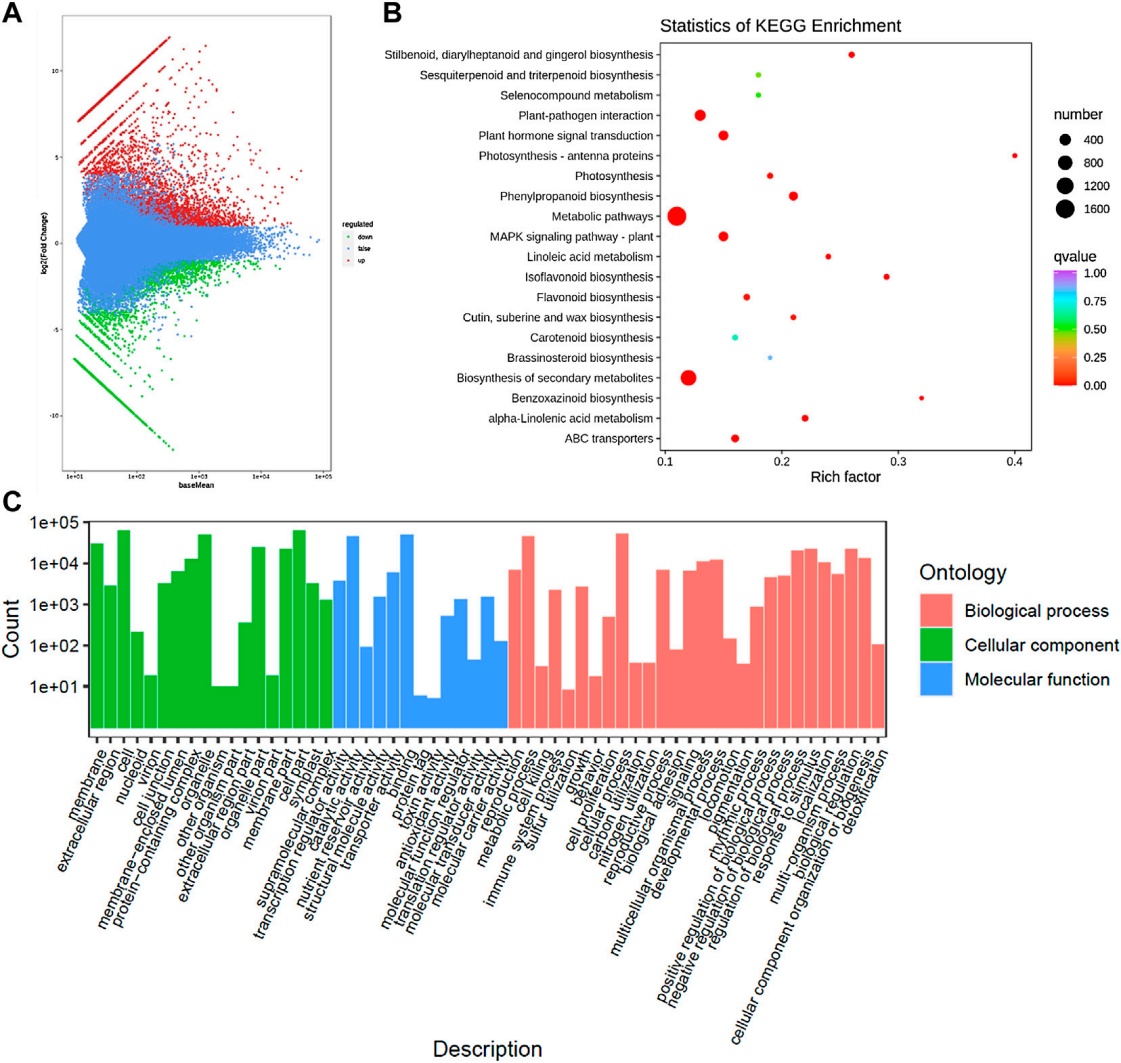
**(A)** Differential gene MA map. The ordinate represents the log2 fold change value; the abscissa represents the average value of gene expression in the two samples; the red dot represents the upregulation of the gene expression, and the green dot represents the downregulation of the expression. Blue indicates no significant difference in gene expression. **(B)** Kyoto encyclopedia of genes and genomes (KEGG) enrichment scatter plot. The ordinate represents the KEGG pathway. The abscissa represents the Rich factor. And **(C)** Histogram of GO classification of the DEGs between CMCs and DDCs.

On the basis of the screening conditions i.e., FDR (<0.05) and log2 foldchange (≥1) 9,564 genes were differentially expressed between CMCs and DDCs (Supplementary Table S2; Figure 3A). Of these, 1,184 and 759 genes were exclusively expressed in DDCs and CMCs, respectively. Overall, 3,713 genes had higher expression while 5,851 had lower expression in CMCs as compared to DDCs. The DEGs were enriched in 137 different KEGG pathways (Supplementary Table S3; Figure 3B). The KEGG classification showed that the DEGs were classified in cellular processes, environmental information processing, genetic information processing, metabolism, and organismal systems. Most DEGs were classified in pathways related to metabolism with the highest number of DEGs (1706) classified in metabolic pathways followed by biosynthesis of secondary metabolites (1,008). In the case of environmental information processing the DEGs were classified in plant hormone signal transduction (245) and MAPK-signaling pathway-plant (235) (Supplementary Figure S1). These observations indicate that the studied cells are experiencing large-scale metabolic changes and hormones and MAPK signaling play essential role in their characteristics.

The exclusively expressed genes in the DDCs could be annotated as 522 KEGG terms. The GO annotation classified the genes into three categories i.e., BP (biological process), CC (cellular component), and molecular function (MF) (Figure 3C). Major BP in which the DEGs were classified included cell prolification, reproduction, cellular component or biogenesis, biological regulation, and regulation of biological processes. The DEGs were classified in GO for 10 MFs; the highest number of DEGs were classified as transcription regulator activity and transporter activities.

### 2.4 Differential Expression of Alkaloid Biosynthesis and Transport Related Genes in Periwinkle CMCs and DDCs

**FIGURE 4:**
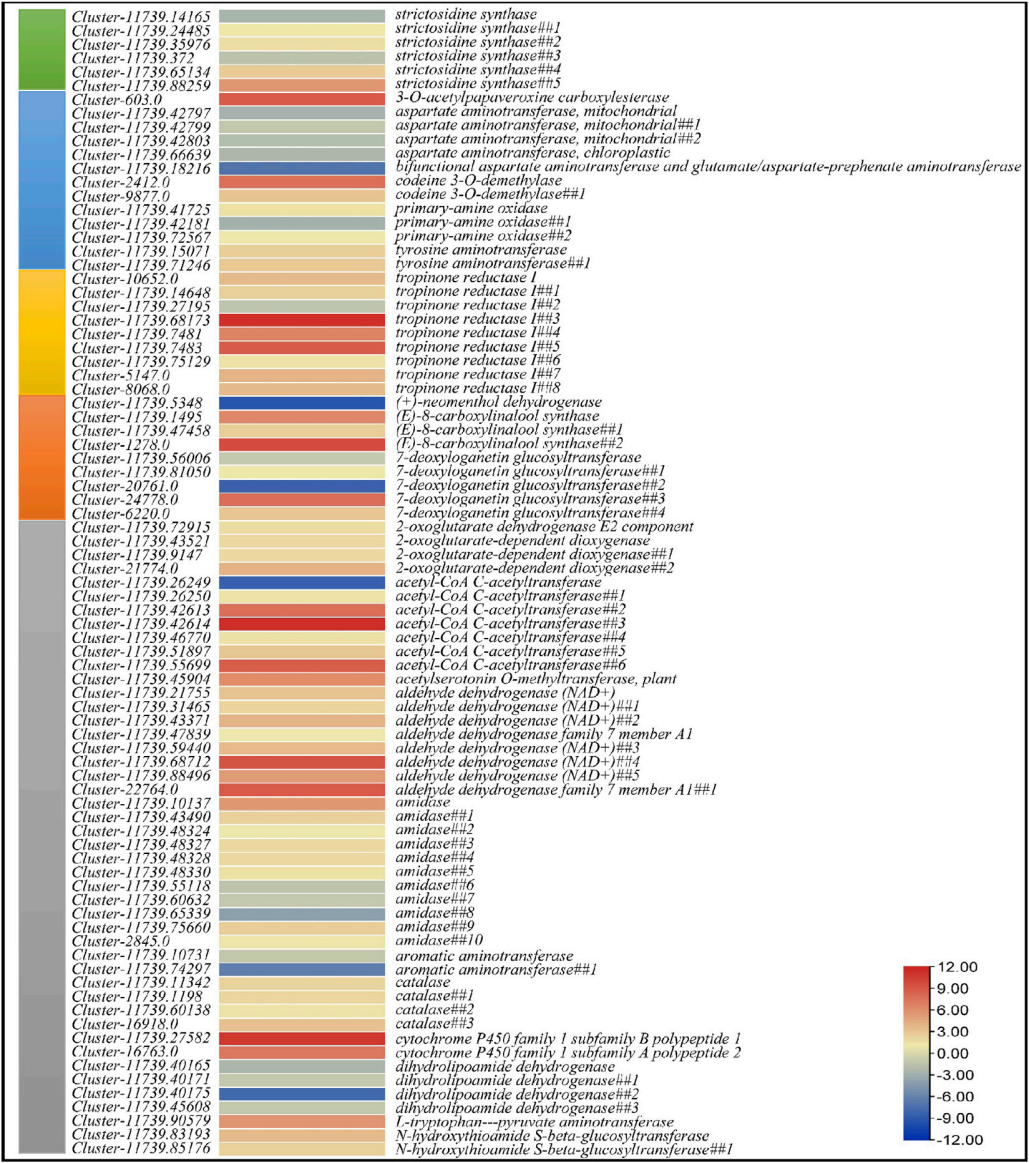
Heatmap of log2 fold change values of the differentially expressed genes in Indole alkaloid biosynthesis (green), Isoquinoline alkaloid biosynthesis (blue), Tropane, piperidine and pyridine alkaloid biosynthesis (dark yellow), Monoterpenoid biosynthesis (orange), and Tryptophan metabolism (grey) pathways.

Since we observed higher content of alkaloids in CMCs as compared to DDCs, we explored the expression of genes that were enriched in isoquinoline alkaloid biosynthesis, Tropane, piperidine and pyridine alkaloid biosynthesis, and Indole alkaloid biosynthesis (as well as the upstream pathways i.e., tryptophan biosynthesis and monoterpenoid biosynthesis). In the indole alkaloid biosynthesis pathway, only six transcripts were differentially regulated which were annotated as strictosidine synthase (Figure 4). Of these, two transcripts i.e., *Cluster-11739.14165* and *Cluster-11739.372* showed reduced expression in DDCs as compared to CMCs suggesting a possible role of these transcripts in the downstream alkaloids’ biosynthesis (Figure 1).

Nineteen DEGs were enriched in tropane, piperidine, and pyridine alkaloid biosynthesis (Figure 4). Four of these were annotated as aspartate aminotransferase mitochondrial (GOT2); all of these had higher expression in CMCs as compared to DDCs. Two transcripts annotated as tyrosine aminotransferase (TAT) showed increased expression in DDCs as compared to CMCs. Rest of the genes i.e., primary-amine oxidases (PAO) and tropinone reductase I (TR1) showed both up- and down-regulation in both types of cells. However, notably only one tropinone reductase I had higher expression in CMCs while rest of the transcripts had lower expression in CMCs as compared to DDCs. Two transcripts (*Cluster-11739.15071* and *Cluster-11739.71246*) annotated as tyrosine aminotransferase showed lower expression in CMCs as compared to DDCs. A bifunctional aspartate aminotransferase and glutamate/aspartate-prephenate aminotransferase (PAT, *Cluster-11739.18216*) was exclusively expressed in CMCs as compared to DDCs. The GOT2, PAT, PAO, and TAT were also enriched in isoquinoline alkaloid biosynthesis pathway. Additionally, two transcripts annotated as codeine 3-O-demethylase (CODM, *Cluster-2412.0* and *Cluster-9877.0*) had lower expression in CMCs as compared to DDCs. The reduced expression of GOT2 in DDCs as compared to CMCs indicates that interconversion of l-phenylalanine and phenylpyruvate is lower in DDCs. This same reaction is also catalyzed by TAT; however, these genes showed lower expression in CMCs as compared to DDCs. These observations indicate that this reaction might be differently regulated in both types of cells (Figure 4).

Since tryptophan biosynthesis and monoterpenoid biosynthesis pathways provide the key molecules that are further processed in indole alkaloid biosynthesis, therefore, we explored the DEGs enriched in these pathways. Forty-six DEGs were enriched in tryptophan metabolism pathway. Of these, 10 DEGs annotated as acetyl-CoA C-acetyltransferase (ACAT1), amidase, aromatic aminotransferase (AroA), and dihydrolipoamide dehydrogenase (DLD) had higher expression in CMCs as compared to DDCs, while the rest of the genes had higher expression in DDCs (Figure 4). Finally, nine DEGs were enriched in monoterpenoid biosynthesis pathway (Figure 4).

There are two types of transporters i.e., H+ antiport carrier system and ABC transporters, involved in intra-cellular alkaloid transport mechanisms (N. Shitan and K. Yazaki, 2007). In this regard, we noted the differential expression of ATP-binding cassette subfamilies A (ABC1), B (MDR/TAP), C (CFTR/MRP), E, and G (WHITE). All ABC1s were highly (solely) expressed in DDCs, whereas, the 1 E member and maximum number of B and G family members were highly expressed in CMCs. The C family had four members up and four downregulated in CMCs as compared to DDCs. For H+ antiport system, we found differential expression of 24 Ca^2+^/H^+^ antiporters (18 of which belonged to TMEM165/GDT1 family) (see orange highlighted gene IDs in Supplementary Table S2). These transporters showed mixed expression i.e., some transcripts were upregulated while others were downregulated in CMCs as compared to DDCs.

### 2.5 Expression Changes in Phytohormone Biosynthetic Genes

Five genes related to auxin biosynthesis were differentially expressed; a tryptophan synthase beta chain (Trp b), a tryptophan---pyruvate aminotransferase (TAA), and three anthranilate synthases (AS). Trp b was exclusively expressed in CMCs, whereas TAA had fractional expression (0.055) in DDCs as compared to CMCs (2.45). The three AS’s were highly expressed in DDCs. These expression changes indicate that anthranilate synthesis is higher in DDCs leading to higher auxin biosynthesis in DDCs, which in turn may negatively regulate TIA biosynthesis. As far as cytokinin biosynthesis is concerned, we noted that 35 of 46 cytokinin biosynthesis related DEGs were highly expressed in CMCs, indicating higher cytokinin biosynthesis in CMCs. The DEGs with higher expression were beta-glucosidases (BGLs), cytokinin dehydrogenase (CDH), cytokinin riboside 5′-monophosphate phosphoribohydrolase (LOG8-like), a CS, cytokinin-N-glucosyltransferases (UGT76C2), cis-zeatin O-glucosyltransferase, cytokinin riboside 5′-monophosphate phosphoribohydrolase, and Arabidopsis histidine kinase 2/3/4. Whereas those with lower expression in CMCs were eight BGLs, a cytokinin synthase (CS), and two Arabidopsis histidine kinase 2/3/4s. Two 1-aminocyclopropane-1-carboxylate synthase-like (ACSs) and three aminocyclopropanecarboxylate oxidases (ACO1s) showed higher expressions in CMCs. However, other ACSs (two ACS3s) and ACOs (ACO1 and ACO3) had contrasting expression patterns. Indicating, ethylene biosynthesis is being regulated by different transcripts in the 2 cell types. We also noted that allene oxide synthases (AOC), and phospholipase A1s (PLA1) had higher expression in CMCs as compared to DDCs, implying higher JA biosynthesis in CMCs. Similarly, seven genes related to SA biosynthesis i.e., isochorismate synthases (ICSs), phenylalanine ammonia-lyases (PALs), and enhanced disease susceptibility one protein (EDS1) showed increased expression in CMCs. However, one of the ICSs (*Cluster-23336.0*) showed DDCs specific expression. Overall, these expression trends indicate higher SA biosynthesis in CMCs (see green highlighted gene IDs in Supplementary Table S2).

### 2.6 Differential Regulation of Plant Hormone Signal Transduction and MAPK Signaling Pathways in Periwinkle CMCs and DDCs

**FIGURE 5:**
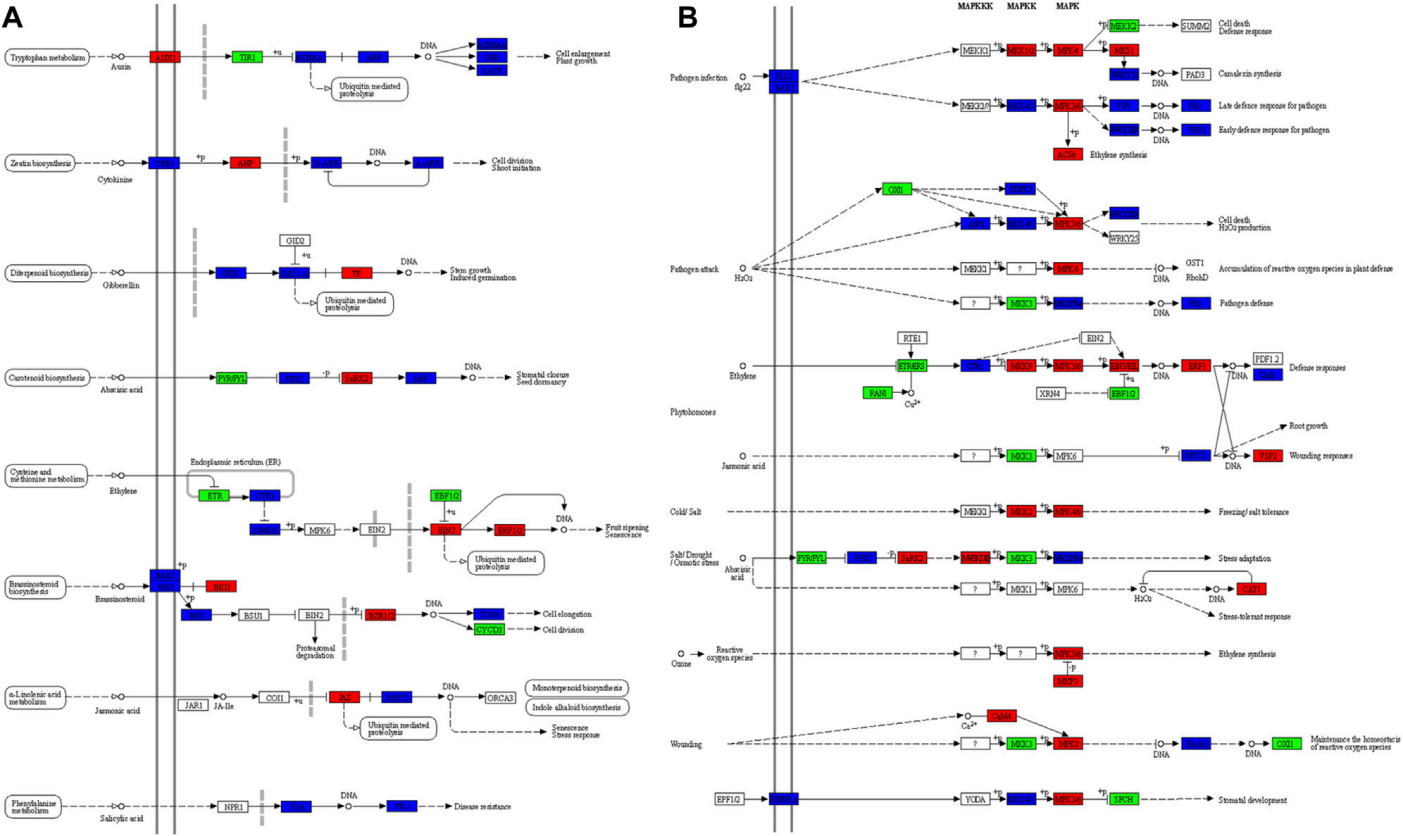
Differently enriched KEGG pathways in *C. roseus* cells (CMCs vs. DDCs). **(A)** Plant hormone signal transduction and **(B)** MAPK signaling-plant pathway. The genes (enzymes) marked in red, green, and blue represent higher, lower, and variable (some transcripts have high and the others have low) expression in DDCs as compared to CMCs, respectively. KEGG maps were developed by using KEGG mapper tool (Kanehisa and Sato, 2020).

A total of 245 and 235 DEGs were enriched in plant hormone signal transduction and MAPK signaling-plant pathways, respectively. Seventy-seven DEGs were common in both pathways. All hormone signaling pathways were differentially regulated in the 2 cell types of *C. roseus.* Most prominently, we observed the higher expression of AUX1 (auxin transporter protein 1), AHP (Arabidopsis histidine phosphotransfer protein), TF (phytochrome interacting factor 4), SnRK2 (serine/threonine-protein kinase SRK2), EIN3 (ethylene-insensitive protein 3), ERF1/2 (ethylene-responsive transcription factor 1), BKI1 (BRI1 kinase inhibitor 1) and BZR1/2 (brassinosteroid resistant 1/2), and JAZ (jasmonate ZIM domain-containing protein) in DDCs as compared to CMCs. On the other hand, TIR1 (transport inhibitor response 1), PYR/PYL (abscisic acid receptor PYR/PYL family), ETR (ethylene receptor) and EBF1/2 (EIN3-binding F-box protein), and CYCE3 (cyclin D3, plant) had higher expression in CMCs as compared to DDCs. The other DEGs showed varied expression in both types of cells i.e., multiple transcripts were expressed. Some had higher expression while others had lower expression in the same type of cells (Figure 5A). Considering that the Jasmonic acid (JA) signaling controls multiple steps upstream of the monoterpenoid biosynthesis and indole alkaloid biosynthesis, the differential expression of JAZ is important. The lower expression of JAZ in CMCs indicates the higher activity of MYC2s as compared to DDCs. However, four MYC2s were exclusively expressed in DDCs, while four others were upregulated in DDCs. Only four MYC2s had higher expression in CMCs as compared to DDCs (Figure 5A). Thus, this step can’t be solely responsible for the observed changes in the indole alkaloid biosynthesis.

Large-scale changes were observed in the MAPK signaling plant pathway (Figure 5B). Specific to JA, we observed that three transcripts annotated as MMK3 (*Cluster-11739.74927*, *Cluster-11739.87079*, and *Cluster-11739.10429*) were highly expressed in CMCs as compared to DDCs. MMK3 is also present upstream the MYC2, thus its importance can’t be negated in terms of the upstream regulation of indole alkaloid biosynthesis. Together with JAZ, the MMK3 genes’ role should be explored in detail in future studies for their effect on the biosynthesis of indole alkaloid biosynthesis.

### 2.7 Other Pathways in Which the DEGs Were Significantly Enriched Between Periwinkle CMCs and DDCs

In addition to the above-described pathways that were related to alkaloid biosynthesis, the KEGG pathway analysis showed the significant enrichment of DEGs in terpenoid backbone biosynthesis, metabolic pathways, biosynthesis of secondary metabolites, flavonoid biosynthesis, phenylpropanoid biosynthesis, and isoflavonoid biosynthesis (Figure 3B). Two of these pathways i.e., metabolic pathways (1,689) and biosynthesis of secondary metabolites (1,008) had a higher number of DEGs enriched in them. These observations indicate that the two types of *C. roseus* cells differ in their metabolic states and secondary metabolites contents. In the case of the phenylpropanoid biosynthesis pathway, the gene [(trans-cinnamate 4-monooxygenase (C4H), *Cluster-11739.25135*)] controlling the conversion of cinnamic acid to p-coumaric acid as well as cinnamoyl-coA to p-Coumaroyl-coA were had higher expression in CMCs as compared to DDCs. Whereas genes annotated as phenylalanine ammonia-lyase (PAL), ferulate-5-hydroxylase (F5H), cinnamyl-alcohol dehydrogenase (CAD), coniferyl-aldehyde dehydrogenase (CALDH), and scopoletin glucosyltransferase (TOGT1) had lower expression in CMCs as compared to DDCs. All other genes showed variable gene expression in both types of cells i.e., some transcripts of the same annotation were expressed higher in one type of cells while other had higher expression on the second type of cells (Supplementary Table S4). The C4H gene was also enriched in flavonoid biosynthesis. Most of the other genes in this pathway showed increased expression in DDCs as compared to CMCs. These observations indicate that CMCs have lower phenylpropanoid and flavonoid biosynthesis as compared to DDCs.

The terpenoid backbone biosynthesis pathway, which is present upstream of the monoterpenoid biosynthesis pathway, was also differentially regulated between CMCs and DDCs. Particularly, 1-deoxy-d-xylulose-5-phosphate reductoisomerase (DXR), (E)-4-hydroxy-3-methylbut-2-enyl-diphosphate synthase (ISPG), and diphosphomevalonate decarboxylase (MVD) had higher expression in CMCs as compared to DDCs. Whereas several other intermediate steps of the terpenoid backbone biosynthesis i.e., between the glycolysis and monoterpenoid biosynthesis were either upregulated in DDCs as compared to CMCs and/or multiple transcripts showed variable expression in both types of *C. roseus* cells (Supplementary Table S4). Since these steps are present upstream of the indole alkaloid biosynthesis pathway, therefore, the genes can be important targets for desired alkaloid production in CMCs.

### 2.8 Differential Expression of Transcription Factors and Transcriptional Regulators in Periwinkle CMCs and DDCs

The comparative transcriptome analysis of CMCs and DDCs indicated differential expression of 529 and 162 transcription factors (TFs) and transcriptional regulators (TRs), respectively. The TFs were classified in 50 different families; 148 and 381 TFs showed increased and decreased expression in CMCs as compared to DDCs, respectively. Of the TRs, 88 and 74 had higher and lower expression in CMCs as compared to DDCs, respectively; the TRs were classified in 19 families of which the most prevalent were SNF2s. The TFs that were exclusively expressed in CMCs were classified as Alfin-like, AP2/ERF, B3, ARF, bHLH, C2H2, C3H, FAR1, HD-ZIP, LOB, MADS-M-type, MYB-related, and NAC; overall 28 TFs were specific to CMCs. The most important observation was the higher expression of CPP, E2F-DP, HB-WOX, OFP, SRS, trihelix, TUB, and whirly TFs in CMCs as compared to DDCs. On the contrary, most other TFs found in our transcriptome sequencing were highly expressed in DDCs as compared to CMCs (Supplementary Table S5). These expression trends indicate that these TFs and TRs can be associated with the changed TIA concentrations in both cell types.

### 2.9 qRT-PCR Based Expression Analysis of Key Genes Involved in Alkaloid Biosynthesis

**FIGURE 6:**
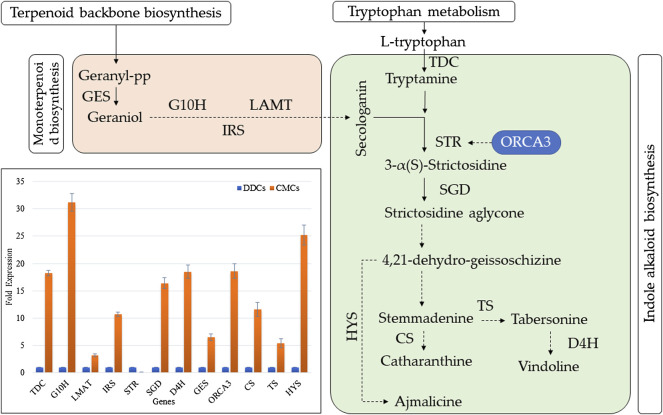
Expression analysis of key genes involved in indole alkaloid biosynthesis in *C. roseus* CMCs and DDCs. TDC (tryptophan decarboxylase); G10H (geraniol 10-hydroxylase (G10H); IRS (iridoid synthase); LMAT (minovincinine 19-O-acetyltransferase); STR (strictosidine synthase); SGD (strictosidine *ß*-glucosidase); D4H (desacetoxyvindoline 4-hydroxylase); GES (geraniol synthase); ORCA3 (Octadecanoid-derivative Responsive Catharanthus AP2-domain); HYS (heteroyohimbine synthase); TS (tabersonine synthase); and CS (catharanthine synthase). The dotted arrows indicate indirect link/uncharacterized reaction/multiple reactions. The normal arrow indicates normal reaction (molecular interaction). The bar graph shows the qRT-PCR based expression of the selected genes. The fold expression is mean of three reactions. The error bars represent standard deviation.

We studied the expression of 12 key genes involved in the biosynthesis of indole alkaloid biosynthesis. The qRT-PCR results showed that the expression of all the studied genes was higher in CMCs as compared to DDCs, except for STR gene (Figure 6). These results are consistent with the higher concentrations of total alkaloids as well as the individual alkaloid i.e., vindoline, catharanthine, ajmalicine, and vinblastine (Figure 2).

## 3 Discussion

Researchers are focusing on industrial scale TIA production using *in vitro* tissue culture techniques e.g., cell suspension cultures. Previous research has shown that CMCs can be distinguished morphologically from DDCs and that the CMCs show stem-cell like properties (Moon et al., 2018). Apart from the distinguishable morphological characteristics, the main benefit of CMCs is their ability to grow faster and maintain homogeneity even after multiple generations. Thus, using the CMCs to produce *in vitro* TIAs can be regarded as useful. The ajmalicine content in CMCs (1.78 mg/g) in our study is higher than those detected in 2-years old hairy root *C. roseus* cultures (0.57 mg/g) (Vázquez-Flota et al., 1994). Also, the cultures from hairy roots didn’t produce vindoline. Comparative to the CMCs in our study, vindoline was the main alkaloid (2 mg/g) in rootless shoots, however, the levels of ajmalicine were low (Hernández-Domínguez et al., 2004). Similarly, the callus culture produced 0.24 mg/g catharanthine, which is higher than our results but the cultures didn’t accumulate any vindoline and ajmalicine (Hernández-Domínguez et al., 2006). Our results that the *C. roseus* CMC cells accumulated sufficient quantities of vindoline catharanthine, ajmalicine, vinblastine, and total alkaloids rather than producing one in higher quantity and lacking others, indicates that these cells don’t produce limits for combined production of these alkaloids (Senbagalakshmi et al., 2017). Therefore, CMCs of *C. roseus* are a better platform for the combined production of these alkaloids as compared to DDCs or the tissues discussed above (Moon et al., 2015; Senbagalakshmi et al., 2017) (Figure 1).

Since these alkaloids are produced in indole alkaloid biosynthesis pathway, therefore, the significant enrichment of DEGs in this KEGG pathway is logical. Furthermore, the differential regulation of upstream pathways sheds light on the key genes that can be manipulated for higher production of these TIAs in CMCs. As a first step in the indole alkaloid biosynthesis pathway (McKnight et al., 1990), the higher expression of two STRs (*Cluster-11739.14165* and *Cluster-11739.372*) indicates a possible reason for the higher TIA biosynthesis in CMCs as compared to DDCs (Supplementary Table S2). Since there were four other transcripts with the same annotation (Supplementary Table S2), therefore, STR cannot explicitly be a possible cause of the observed changes in the downstream alkaloid contents, the varied expression of STRs is also consistent with qRT-PCR results of STR as well as ORCA. Meanwhile, we didn’t detect differential expression of ORCA in CMCs vs. DDCs, therefore we cannot strictly conclude about its role in our experimental material. However, the strong expression of ORCA recorded in QRT-PCR analysis can be due to the differential expression of JA and/or ethylene biosynthesis and signaling related genes. This statement is based on a recent report that ORCAs show significant variation in expression in response to MeJA and ethylene (Singh et al., 2020). Also, the ORCA group in *C. roseus* also include AP2/ERFs, therefore, their exclusive expression in CMCs can be an explanation for higher TIA biosynthesis (Singh et al., 2020). Going further upstream, two key pathways i.e., monoterpenoid biosynthesis and tryptophan metabolism showed that there are more reaction steps that can be regarded as important for differential TIA biosynthesis in CMCs and DDCs (Supplementary Tables S2–4). In tryptophan metabolism, the AroA gene converts indolepyruvate to tryptophan (Kradolfer et al., 1982). While DLD converts 2-Oxoadipate to glutaryl-CoA (Otulakowski and Robinson, 1987). The earlier step can be considered the key step that might play important role in the biosynthesis of the studied alkaloids. While the one controlled by DLD cannot be directly linked with the observed changes in the concentration of alkaloids. These expression changes and the qRT-PCR showing that CMCs had higher TDC expression indicate that the conversion of tryptophan to tryptamine is higher in CMCs as compared to DDCs. Previous work in *C. roseus* has shown that the overexpression of STR and TDC genes resulted in higher TIA biosynthesis (Canel et al., 1998), which correlates with our current observations. Tryptamine is further converted into secologanin and ultimately produced TIAs. Another pathway that should be discussed here is the monoterpenoid biosynthesis which also yields secologanin for TIA biosynthesis (Oudin et al., 2007). One key step in this pathway i.e., the conversion of deoxyloganetin to 7-deoxyloganin can be regarded as important upstream reaction for the alkaloid biosynthesis. This step is catalyzed by 7-deoxyloganetin glucosyltransferase (UGT85A23) (Asada et al., 2013); five DEGs were annotated as UGT85A23. However, the expression of one of these transcripts (*Cluster-20761.0*) is notable since it was exclusively expressed in CMCs. However, another one (*Cluster-24778.0*) was exclusively expressed in DDCs. Thus, this step can’t be the only possible reason for higher alkaloid biosynthesis in CMCs (Figure 5). Moving upstream, the higher expression patterns of DXR and ISPG genes in terpenoid biosynthesis pathway hint that in CMCs the increased TIA biosynthesis is being controlled at the various steps in multiple pathways. It also indicates that CMCs somehow trigger the expression of the above discussed genes to overall increase the key compounds’ concentrations in order to produce higher TIAs. These results are consistent with the qRT-PCR analysis of GES, G10H, IRS, and LAMT genes (Figure 5). In a previous gene to metabolite study in *C. roseus* cells Rischer, et al. (Rischer et al., 2006) a similar conclusion was made. Our study specifically directs that CMCs should be exploited at these reaction steps to yield higher quantities of TIAs as compared to DDCs.

Contrastingly, we know that vindoline and bisindole alkaloid biosynthetic potential is different between plant tissues, cell cultures, and suspension cultures. Among these, the cell suspension cultures have been reported to have least biosynthetic potential. These changes are associated with two major things. First, the process of differentiation results in differential alkaloid levels and second is the elicitation with different phytohormones and/or fungal homogenates (Shukla et al., 2010). In *C. roseus,* it has been established that differentiation and redifferentiation can cause changes in the ability of the cultures to produce specific alkaloids. For example, cell cultures that cannot produce vindoline can be recovered for this defect after redifferentiation [ (St-Pierre et al., 1999) and references therein]. However, in our study our specific focus was not the developmental difference between the two types of cells but key TIA related transcriptomic signatures. In this regard, our observation that five of eight deacetylvindoline 4-O-acetyltransferase (DATs) had exclusive/higher expression in CMCs (Supplementary Table S2), can be associated with vindoline biosynthesis. This is consistent with earlier report in *C. roseus* (Campos-Tamayo et al., 2008)*.* This could also be due to the differentiation of the 2 cell types but we didn’t further explore this particular question and hence further studies would elaborate it more.

From transport perspective, the higher expression of a large number of B subfamily members in CMCs indicates increased activities such as mediating auxin transport and translocation of alkaloids in CMCs. Previously it is known that ABCB1 members in Arabidopsis mediate auxin efflux (Kim et al., 2010). Specifically, the study found that ABCB member inhibited auxin transport (Kim et al., 2010). Thus, higher expression in CMCs could have helped them avoid negative effects of auxin (Yang and Murphy, 2009). Similarly, the expression of a *Coptis japonica* MDR1 in *C. roseus* suspension cells resulted in increased accumulation of ajmalicine and tetrahydroalstonine. Therefore, their higher expression and increased accumulation of TIAs (particularly ajmalicine) in CMCs is relatable. Similarly, the higher expression of G sub family transcripts is in line with the higher accumulation of TIAs and known role of ABCGs i.e., a *CrTPT2* lead enhanced catharanthine accumulation (Yu and De Luca, 2013). Furthermore, the transcript expressions of Ca^2+^/H^+^ antiporters indicate active proton-driven vacuolar transport of vindoline and catharanthine (Schumaker and Sze, 1985; Carqueijeiro et al., 2013).

Terpenoid indole alkaloid biosynthesis has been studied for the past 4 decades and a wealth of knowledge has shown that their biosynthesis is affected by multiple factors i.e., light, plant growth regulators (phytohormones), and signaling molecules [3 and references therein]. The fact that we observed the enrichment of DEGs in plant-hormone signaling and MAPK signaling pathways (Figure 5) in our comparative transcriptome analysis is consistent with the earlier reports (Zhu et al., 2015; Matsuura et al., 2014; Zhou et al., 2010). In phytohormones, auxins negatively regulate the biosynthesis of TIAs. Thus, the contrasting expression patterns of AUX1s and TIR1s in DDCs can be related to this known negative regulation and the observed lower TIA biosynthesis as compared to CMCs (Pasquali et al., 1992). On the contrary, cytokinins are known for their roles in remarkable increases in alkaloid biosynthesis in *C. roseus* (Papon et al., 2005), by enhancing the expression of G10H gene (whose expression was higher in CMCs as compared to DDCs, Figure 6). The higher expression of AHP (*Cluster-11739.19941* and *Cluster-11739.27783*) transcripts in DDCs as compared to CMCs can also be regarded as a key regulatory step since AHPs have been implicated in the inhibition of cytokinin signals. Furthermore, the higher expression of 35 cytokinin biosynthetic genes clearly indicates, higher cytokinin biosynthesis in CMCs. Which resultantly induce TIA biosynthesis (Papon et al., 2005). Other than this, the higher expression of ACS and ACOs in CMCs indicates their roles in higher TIA biosynthesis in CMCs. This proposition is based on the known roles of ACS, ACO, G10H, and AHPs (Miyata et al., 1998; Pattyn et al., 2021). Similarly, Jasmonates and SA have been reported as signaling molecules that lead to the higher biosynthesis of ajmalicine (Rijhwani and Shanks, 1998), vincristine (Idrees et al., 2011), vinblastine (Idrees et al., 2011), and vindoline (El-Sayed and Verpoorte, 2004). Our results for higher accumulation of these TIAs in CMCs are consistent with these reports since respective phytohormone biosynthetic genes had higher expressions in CMCs.

Not only the phytohormone signaling but MAPK signaling pathway related genes have also been characterized in *C. roseus* for their effects on monoterpenoid indole alkaloids biosynthesis. For example, a mitogen-activated protein kinase i.e., CrMPK3 was activated under the influence of JA, ultraviolet radiations, and wounding, resulting in higher indole alkaloid biosynthesis (Raina et al., 2012). The higher expression of MKK3 in CMCs indicates that the MKK3-MKK6 and MYC2 module have some functional role in JA signaling in these types of *C. roseus* cells. However, the MKK6 (a part of this module (Sethi et al., 2014)) didn’t differentially express, and the fact that multiple MYC2s had variable expression patterns in both types of cells (Supplementary Table S3) calls for detailed characterization of this module in the presence/absence of certain factors (e.g., light and/or wounding) in both types of *C. roseus* cells. At this stage it is premature to assume any role of JA (or its signaling based on transcriptomic signatures) on the differential TIA biosynthesis in CMCs and DDCs (Goldhaber-Pasillas et al., 2014). Taken together, these transcriptomic changes propose that CMCs have ability to produce higher TIA levels as compared to DDCs under the influence of plant-hormone signaling and MAPK signaling pathways.

Finally, the exclusive expression of bHLH, AP2/ERF, and MYB-related TFs is consistent with the TIA synthetic genes (Figure 6). These observations are consistent with the earlier studies where bHLH gene in *Medicago truncatula* caused up-regulation of triterpene saponins (Mertens et al., 2016). Similarly, the overexpression of AP2/ERF in tobacco (Mishra et al., 2015)and MYBs in *Artemisia annua* (Yu et al., 2012) and cabbage (Kim et al., 2013) could increase TIA biosynthesis. However, the roles of other TFs for whom we found higher/exclusive expressions in CMCs are not yet characterized. Therefore, this transcriptome also presents a number of novel TF candidates whos expressions can be associated with higher/lower TIA biosynthesis in *C. roseus* cells. Although tens of studies have defined roles of a range of TF families in TIA biosynthesis, but still there is a long way forward (Liu et al., 2016).

## 4 Materials and Methods

### 4.1 Plant Material

**FIGURE 7:**
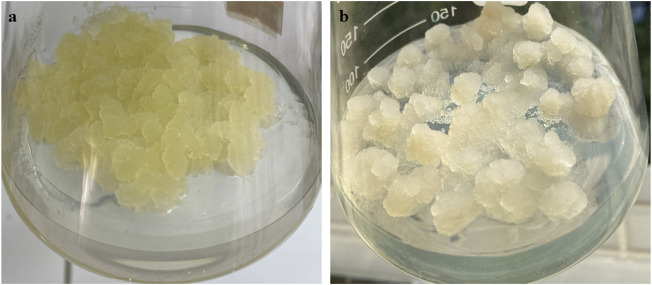
The samples of 12-day-old **(A)** cambial meristematic cells (CMCs) and **(B)** dedifferentiated cells (DDCs) *C. roseus* that were used for the extraction of RNA extraction for transcriptome sequencing comparisons.

The dedifferentiated cells [(DDCs) (CAT-1)] of *C. roseus* were established according to the modified method of Zhou et al., (Zhou et al., 2015). Whereas, the cambial meristematic cells [(CMCs) (CAT-2] are re-induced establishments of vinblastine containing CMCs reported in Zhou et al., (Zhou et al., 2015) (Figure 7). Briefly, twigs were collected from *C. roseus* growing in wild, rinsed in the running tap water for 30 min, disinfection with 75% ethanol for 30 s, and rinsing five times with sterilized distilled water (dH_2_O). The disinfected twigs were then put in 0.05% HgCl_2_ for 6 min, rinsing with dH_2_O five times, followed by rinsing with 150 mg/L citric acid (dissolved in dH_2_O). Cambium, phloem, cortex, and epidermal tissues were peeled from the xylem, and the epidermal tissue was placed on pH-adjusted (5.75) Murashige and Skoog (MS) medium (Murasnige and Skoog, 1962) for 24 days. The MS medium was supplemented with 1.0 mg/L *a*-naphthalene acetic acid (NAA), 30 g/L sucrose, and 4 g/L gelrite. After 24 days, the cambial cells formed a flat plate while phloem, cortex, and *epidermis* originated DDCs proliferated in irregular fashion. Cambial cells were separated, transferred to 250 ml Erlenmeyer flasks containing 100 ml of MS medium supplemented with 2.0 mg/L NAA, 10 g/L sucrose, and 4 g/L gelrite. The CMCs were cultured at 25°C under continuous dark and were maintained by serial subculture every 12 days. Two months prior to the experiments, CMCs were transferred to 100 ml of MS solid medium in a 250-ml Erlenmeyer flask and grown at 25°C with a 12/12-h light/dark photoperiod. Since cells were sub-cultured for every 12th day, therefore after 6 months, the cells used for the alkaloid analyses were 13th generation.

For determining growth rates, CMCs and DDCs were subcultured every 12 days. CMCs or DDCs (5.0 g fresh weight) were transferred to 250-ml Erlenmeyer flasks respectively, containing 100-ml MS liquid medium supplemented with 2.0 mg/L NAA and 30 g/L sucrose. Suspension cultures of *C. roseus* CMCs and DDCs were incubated on a HZT-2 gyrotory shaker (Donglian Electronic and Technol. Dev. Co., Beijing, China) at 25°C and 120 rpm under continuous light. Cultures were harvested after 3, 6, 9, 12, 15, 18 days. Cell growth was determined by grams of dry weight (DW) per liter as reported earlier (Zhou et al., 2015).

### 4.2 Biochemical Analyses

The total alkaloid content was estimated using HPLC by following the method described by Harborne (Harborne, 1998). We also measure the concentrations of vindoline, catharanthine, ajmalicine, and vinblastine in the CMCs and DDCs. For this, first we isolated these compounds following the method described by Schripsema and Verpoorte (Schripsema and Verpoorte, 1992). The extracts were filtered and analyzed by HPLC. The separation of the four compounds was achieved by using Diamonsil C18 column (250 mm × 4.6 mm, 5 μm), by using 380 ml solution A [water: diethylamide = 986:14 (v/v), pH 7.2] combined with 620 ml solution B [methanol: acetonitrile = 4:1 (v/v)] as the mobile phase. The detection was performed at a flow rate of 1.5 ml min-1 and a wavelength of 220 nm within 40 min as described earlier (Hashemabadi et al., 2018).

### 4.3 Transcriptome Analysis

#### 4.3.1 RNA Extraction, Library Preparation, and on Machine Sequencing

High quality RNA was extracted from the CMC and DDC in triplicate by using Spin Column Plant total RNA Purification Kit (Sangon Biotech, Shanghai, China) following the manufacturer’s instructions. The quality of the extracted RNAs from the six samples was assessed by agarose gel electrophoresis (integrity of RNA and detection of DNA contamination), NanoPhotometer (RNA purity), Agilent 2,100 bioanalyzer (accurately detect RNA integrity). Once the purity and integrity of RNA was confirmed, we purified mRNA from the total RNAs by using poly-T-attached magnetic beads and then used fragmentation buffer to break the RNA into short fragments. cDNA was synthesized from the short RNA fragments by using cDNA synthesis kit (ThermoFisher, Scientific, United States). The double-stranded cDNA was then purified using AMPure XP beads, repaired, A-tailed, and ligated with a sequencing adapter. We then used AMPure XP beads to fragment size selection followed by PCR enrichment to obtain a final cDNA library. Quality of the library was tested for insert size detection by Qubit 2.0 and Agilent 2,100 bioanalyzer. A Q-PCR was performed to determine the effective library concentration (>2 nM) followed by sequencing of the prepared libraries on Illumina HiSeq platform (Illumina Inc., San Diego, CA, United States).

### 4.4 Computational Analyses of RNA-Seq Data

Before analyses, the raw sequencing data was checked for quality control by removing reads (having adaptors, paired reads having >10% N content, and if the low-quality basis Q ≤ 20 was >50%). We then determined the error distribution and GC content in the sequencing reads. We then used BLAST (Johnson et al., 2008) to compare the unigene sequences with KEGG (Kanehisa and Goto, 2000), NR (Deng et al., 2006), Swiss-Prot (Apweiler, 2001), GO (Ashburner et al., 2000), COG/KOG (Koonin et al., 2004), Trembl databases (Apweiler et al., 2004). Furthermore, we predicted unigenes’ amino acid sequences and used HMMER software to compare the sequences with Pfam (Bateman et al., 2002). The gene expression was quantified by splicing the transcripts by Trinity (ref. sequence) and then map the clean reads to each ref. sequence by using bowtie2 (Langmead and Salzberg, 2012) in RSEM (Li and Dewey, 2011). The Fragments Per Kilobase of transcripts per Million fragments mapped (FPKM) was calculated and visualized in R. Pearson Correlation Coefficient (PCC) and Principal Component Analysis (PCA) were done on the gene expression data in R. The differentially gene screening between the CMC and DDC was done in DESeq2 (Varet et al., 2016). Then Benjamini–Hochberg method (Benjamini and Hochberg, 1995) was used to perform hypothesis test correction on *p*-value to obtain false discovery rate (FDR) and screen the differentially expressed genes (DEGs) with the conditions that if FDR (<0.05) and log2 foldchange (≥1), the gene was differentially expressed. After identifying DEGs, they were enriched in KEGG pathways in KOBAS2.0; care was taken to reduce false positives for the prediction of KEGG pathways by considering FDR <0.05. Finally, we used iTAK software to predict plant transcription factors (TF); it integrates PlnTFDB and PlantTFDB and uses TF family and identifies TF through HMM-HMM scan comparison (Zheng et al., 2016).

### 4.5 qRT-PCR Analysis

We studied the expression of 12 genes that are associated with the TIA biosynthesis pathway (Table 1). First strand cDNAs were synthesized from 100 ng of total RNA using the High-Capacity cDNA Reverse Transcription Kit (Applied Biosystem, United States). The primers for each gene are given in Table 1. The reactions were carried out in 20 μl each on a Rotor-Gene 6,000 machine (Qiagen, Shanghai, China). 40S Ribosomal protein S9 (RPS9) was used as a single reference gene (Menke et al., 1999). The reaction mixture contained the following amounts of the reagents. SYBR ^®^ Premix Ex Taq (Tli RNaseH Plus) (2x) 10. µL, forward primer (5 Mm) 0.8 µl, reverse primer (5 Mm) 0.8 µl, DNA template 2.0 µl, RNase Free dH2O 6.4 µl. The reactions conditions were as follows. Pre-denaturation at 95°C for 30 s, 20°C/s (1 cycle), 40 cycles of 95°C for 5s, 60°C for 20s and 20°C/s, followed by the melting curve analysis at 95°C for 0 s, 20°C/s, 65°C for 15s, 20°C/s, 95°C for 0 s, and 0.1°C/s. For each sample, two biological replicates were analyzed in independent runs.

**TABLE 1:** Primers for qRT-PCR analyses of *C. roseus* reference and TIA biosynthetic genes.

Gene	Primer Sequence
RPS9 (40S Ribosomal protein S9)	F = TGA​AGC​CCT​TTT​GAG​GAG​GAT​G
	R = TGC​CAT​CCC​AGA​CTT​GAA​AAC​A
LAMT (Loganic acid methyltransferase)	F = GAG​TAA​TTG​ATG​CAG​CCA​AG
	R = TTG​ATT​GGA​TCA​AAG​ATT​GG
IRS (Iridoid synthase)	F = CCT​AGG​CTA​AAT​GTC​CCA​AA
	R = GTC​TAT​GGA​CAG​ACC​ATG​TT
TDC (Tryptophan decarboxylase)	F = TCC​GAA​AAC​AAG​CCC​ATC​GT
	R = AAG​GAG​CGG​TTT​CGG​GGA​TA
G10H (Geraniol 10-hydroxylase)	F = TGA​ATG​CTT​GGG​CAA​TTG​GA
	R = GCA​AAT​TCT​TCG​GCC​AGC​AC
STR (Strictosidine synthase)	F = TGA​CAG​TCC​CGA​AGG​TGT​GG
	R = CGC​CGG​GAA​CAT​GTA​GCT​CT
SGD (Strictosidine *ß*-glucosidase)	F = ATG​AGA​GCT​CTT​GTA​GGA​AGC​CGT
	R = GCG​CAC​TTC​CTT​CCC​ATC​AAC​TTT
D4H (Desacetoxyvindoline 4-hydroxylase)	F = TAC​CCT​GCA​TGC​CCT​CAA​CC
	R = TTG​AAG​GCC​GCC​AAT​TTG​AT
GES (Geraniol synthase)	F = TTGTTTTCGATTGCTTCG
	R = TCT​ATG​TCT​TGG​TTG​CTC​TA
ORCA3 (Octadecanoid-derivative Responsive *Catharanthus* AP2-domain)	F = CGA​ATT​CAA​TGG​CGG​AAA​GC
	R = CCT​TAT​CTC​CGC​CGC​GAA​CT
CS (Catharanthine synthase)	F = CTC​CTG​GCG​GGA​TGA​ATA​AC
	R = GGA​AAC​CAG​GGT​AAC​CAA​CA
TS (tabersonine synthase)	F = AGA​TGC​TCC​TGG​TGG​AAA​TG
	R = CAA​CCA​TGG​AAA​TCA​GCA​ACC
HYS (heteroyohimbine synthase)	F = AGC​AAT​CAG​ATT​TGC​CAA​GG
	R = GGG​TTA​CTG​TTG​AGC​AAG​AAA​G

## 5 Conclusion

We compared to total alkaloid, vinblastine, vindoline, catharanthine, and ajmalicine contents in two types of *C. roseus* cell suspension cultures i.e., CMCs and DDCs. We conclude that CMCs have potential to produce higher quantities of these alkaloids. The DDCs were unable to produce (undetectable in our study) vinblastine and vincristine. Using Illumina HiSeq sequencing platform, we sequenced the transcriptome of *C. roseus* CMCs and DDCs. KEGG pathway enrichment analysis concluded that the studied cells have large scale metabolic changes between them and hormones and MAPK signaling play essential role in their characteristics. The differential expression in the key genes in indole alkaloid biosynthesis pathway, tryptophan metabolism, monoterpenoid biosynthesis, and terpenoid backbone biosynthesis pathways can be correlated with the observed differences in the total alkaloid, vinblastine, vindoline, catharanthine, and ajmalicine biosynthesis.

## Data Availability

The datasets presented in this study can be found in online repositories. The names of the repository/repositories and accession number(s) can be found in the article/Supplementary Material.
