# Supplementary figures and images for: Exploration of the Mechanisms of Differential Indole Alkaloid Biosynthesis in Dedifferentiated and Cambial Meristematic Cells of Catharanthus roseus Using Transcriptome Sequencing
Source: Front Genet. 2022 Jun 30;13:867064. doi: 10.3389/fgene.2022.867064 (PMC9305860; doi:10.3389/fgene.2022.867064)

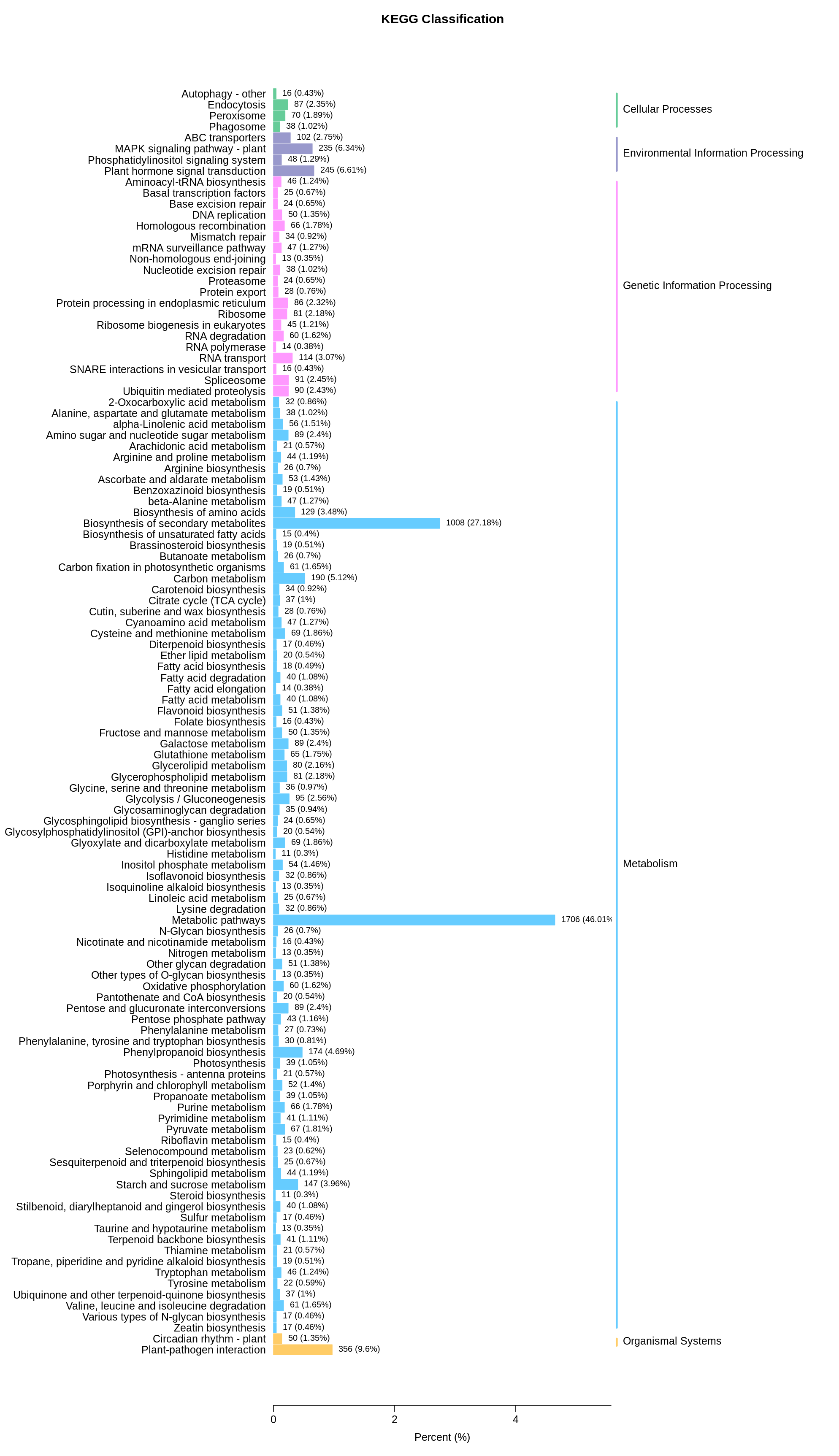

Supplement: Supplementary file 2 [file Image1.PNG]
